# Hypothermal opto-thermophoretic tweezers

**DOI:** 10.21203/rs.3.rs-2389570/v1

**Published:** 2023-01-20

**Authors:** Pavana Siddhartha Kollipara, Xiuying Li, Jingang Li, Zhihan Chen, Hongru Ding, Suichu Huang, Zhenpeng Qin, Yuebing Zheng

**Affiliations:** 1 Walker Department of Mechanical Engineering, The University of Texas at Austin, Austin, Texas, 78712, USA.; 2 Department of Mechanical Engineering, The University of Texas at Dallas, Richardson, Texas, 75080, USA; 3 Materials Science and Engineering Program and Texas Materials Institute, The University of Texas at Austin, Texas, 78712, USA; 4 Laser Thermal Laboratory, Department of Mechanical Engineering, University of California, Berkeley, California 94720, USA; 5 Key Laboratory of Micro-Systems and Micro-Structures Manufacturing of Ministry of Education and School of Mechatronics Engineering, Harbin Institute of Technology, Harbin 15001, China; 6 Department of Bioengineering, The University of Texas at Dallas, Richardson, Texas, 75080, USA; 7 Department of Surgery, The University of Texas Southwestern Medical Center, Dallas, Texas, 75390, USA; 8 Center for Advanced Pain Studies, The University of Texas at Dallas, Richardson, Texas, 75080, USA

## Abstract

Optical tweezers have profound importance across fields ranging from manufacturing to biotechnology. However, the requirement of refractive index contrast and high laser power results in potential photon and thermal damage to the trapped objects, such as nanoparticles and biological cells. Optothermal tweezers have been developed to trap particles and biological cells via opto-thermophoresis with much lower laser powers. However, the intense laser heating and stringent requirement of the solution environment prevent their use for general biological applications. Here, we propose hypothermal opto-thermophoretic tweezers (HOTTs) to achieve low-power trapping of diverse colloids and biological cells in their native fluids. HOTTs exploit an environmental cooling strategy to simultaneously enhance the thermophoretic trapping force at sub-ambient temperatures and suppress the thermal damage to target objects. We further apply HOTTs to demonstrate the three-dimensional manipulation of functional plasmonic vesicles for controlled cargo delivery. With their noninvasiveness and versatile capabilities, HOTTs present a promising tool for fundamental studies and practical applications in materials science and biotechnology.

## Introduction

The development of optical tweezers has led to tremendous advances in many fields such as optical nanomanufacturing,^1^ microrobotics,^2^ cell mechanics,^3^ and nanomedicine.^4^ Optical tweezers trap target objects by the gradient force, which depends on the refractive index, particle size, and laser wavelength.^5^ High laser power is usually required to trap nanomaterials and biological objects that have a low refractive index contrast with their surroundings, which can induce damage to the materials and reduce the cell viability.^6^

To overcome these challenges, different variations of optical tweezers, such as plasmonic tweezers,^7–10^ opto-electronic tweezers,^11,12^ and opto-acoustic tweezers,^13^ have been developed. However, they are usually limited by specific substrates, complex setups, or confined working ranges. Recently, optothermal tweezers have been developed to achieve versatile manipulation of colloidal particles under a light-controlled temperature gradient^14–18^. While optothermal tweezers enable enhanced trapping capability with a laser power that is 2–3 orders of magnitude lower than optical tweezers,^19^ optical heating can cause thermal stress and degradation to the particles and biological cells. In addition, since many colloids and cells show a thermophobic behavior and move away from the laser heating spot, optothermal tweezers require additional surfactants or salts to tune their thermophoretic response.^20–23^ However, trapping cells and biological objects in required fluidic environments is often essential for biological applications to reduce the effect of additives and elucidate the bio-physio-chemical interactions of the cells.^24–26^

In this work, we propose hypothermal opto-thermophoretic tweezers (HOTTs) to overcome these limitations. Specifically, we couple environmental cooling and localized laser heating to achieve low power thermophoretic trapping of target objects and simultaneously avoid optical and thermal damage. More importantly, this cooling strategy also plays a vital role in facilitating the thermophilic behavior to enable the trapping of diverse colloids at different conditions. We also demonstrate the successful trapping and manipulation of fragile erythrocytes in different tonicities to resemble different bio-physio-chemical functionalities. We further show the capability of HOTTs for three-dimensional manipulation (3D) of plasmonic vesicles for light-controlled drug delivery.

## Working Principle of HOTTs

**Figure 1:**
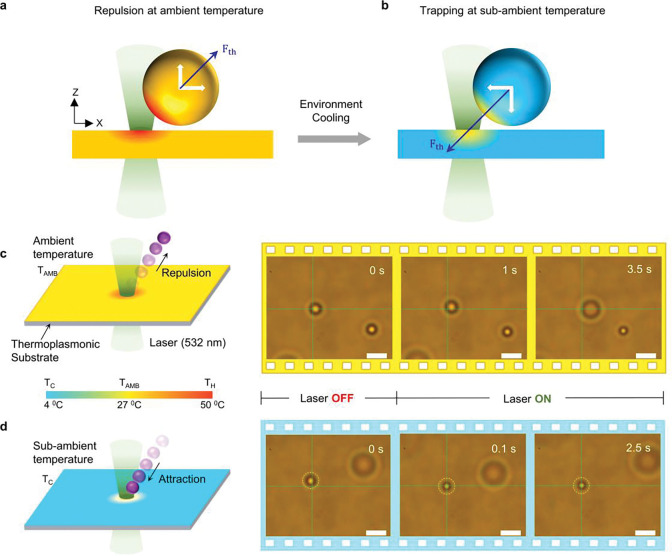
Working principle of HOTTs: a) At ambient temperature, thermophoretic force (*F*_*th*_) repels the particle away from the laser in most conditions. White arrows indicate *F*_*th*_ decomposed along and perpendicular to the substrate b) In HOTTs, *F*_*th*_ becomes attractive to trap particles at a sub-ambient temperature. c) Schematic and timelapse optical images showing the repelling of a 1 *μ*m PS particle in DI water by the laser beam at an ambient temperature of 27 °C d) The same particle was trapped at the laser beam at a sub-ambient temperature of 4 °C. The green crosshair indicates the laser beam center. Laser wavelength: 532 nm, laser power: 40 *μ*W, beam radius: 850 nm, scale bars: 2 *μ*m.

Figs. 1a and 1b depict the general schemes of opto-thermophoretic trapping at ambient temperature and under environmental cooling, respectively. A thermoplasmonic substrate is used to generate a temperate gradient (∇*T*) under local laser heating (see Methods). The particle under the temperature gradient is subject to a thermophoretic force (*F*_th_),^20, 27^ which can be expressed as

(1) $$F_{\text{th}}=-k_{B}TS_{T}\nabla T$$

where *S*_*T*_ is the Soret coefficient of the particle, *k*_*B*_ is the Boltzmann constant, and *T* is the average temperature around the particle (see Supplementary Note 1). The direction of thermophoretic force is dependent on the sign of *S*_*T*_ and a positive (or negative) *S*_*T*_ leads to a repulsive (or attractive) force. *S*_*T*_ is a function of many parameters, including colloid composition, ionic concentrations, surface effects, particle size, and temperature.^28,29^ *S*_*T*_ decreases with the decreasing temperature in the majority cases, which can be described by an empirical equation^30^

(2) $$S_{T}(T)=S_{T,\infty }\left(1-e^{\frac{T^{*}-T}{T_{0}}}\right)$$

where *S*_*T*,∞_ is the high-temperature limit, T* is the transition temperature where *S*_*T*_ changes the sign, and *T*_0_ represents the strength of the temperature effect. At the ambient temperature, *S*_*T*_ is positive for most objects and the thermophoretic force repels them away from the laser (Figure 1a). In HOTTs, we adopt an environment-cooling strategy to enable a negative *S*_*T*_ and a thermophoretic attractive force to trap the particle at the hotspot (Figure 1b). A custom temperature controller based on Peltier cooling is designed to enable fast cooling of the sample (Figure S1 and Supplementary Note 2). As a proof-of-concept demonstration, we compared the trapping behaviors of 1 *μ*m polystyrene (PS) microparticle in deionized (DI) water at two different conditions. At the ambient temperature of 27 °C, the PS microparticle was repelled away from the laser beam due to a net repulsive thermophoretic force (Figure 1c). In contrast, when the environmental temperature was cooled down to 4 °C, the particle was successfully trapped at the laser beam center by the thermophoretic attraction force (Figure 1d and Supplementary Video 1).

## Versatility of HOTTs

**Figure 2:**
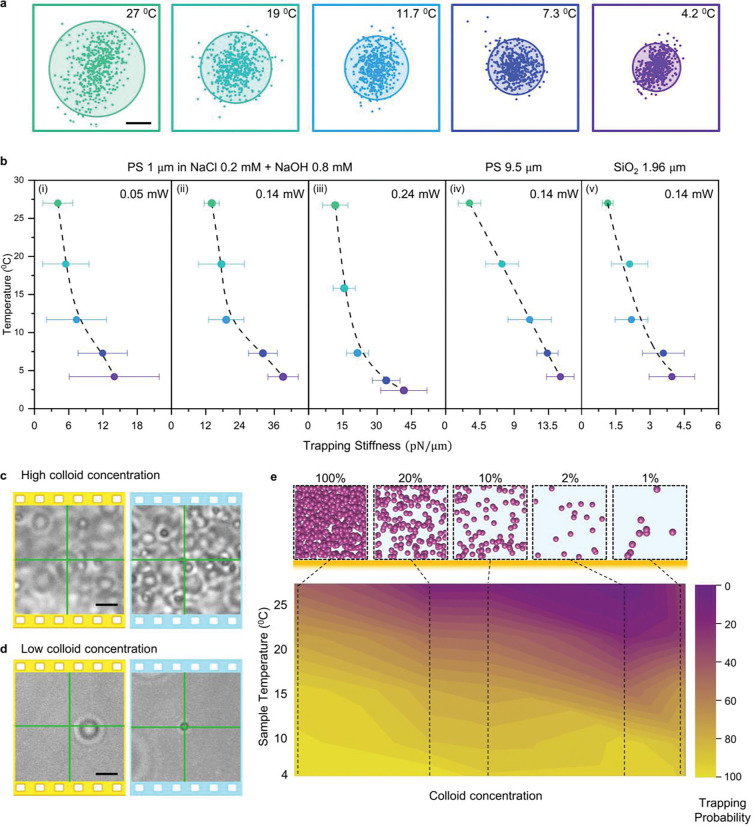
Evaluation of trapping performance of microparticles using HOTTs: a) Particle trajectories of 1 *μ*m PS particles (low colloid concentration) in 1mM NaCl_0.2_OH_0.8_ solution at varying environmental temperature. b) Trapping stiffness dependence on sample temperature at single-particle concentration indicates the enhancement of trapping efficiency at lower temperatures for varying laser powers (i-iii), sizes (ii, iv), materials (ii, v), and solutions (i-iii, iv-v). c,d) Optical images of repulsion at 27 °C (yellow panels) and trapping at 4 °C (blue panels) of 1 *μ*m PS particle in DI water at a high concentration of 28.6 mg/mL (c) and low concentration (d) of 0.29 mg/mL. Laser power is 50 *μ*W. e) Trapping probability of 1 *μ*m PS particles as a function of sample temperature and colloidal concentration. The highest concentration of 1 *μ*m PS particles at 100% relative concentration is 28.6 mg/mL. Laser power is 50 *μ*W. Scale bars: (a) 1 *μ*m (c,d) 5 *μ*m.

Next, we demonstrate the use of HOTTs to trap diverse microparticles in different conditions to demonstrate its wide applicability (Figure 2, Figure S2, and Supplementary Video 2–4). In all cases, thermophoretic trapping of colloids (e.g., PS and silica microparticles) is enabled or significantly enhanced in HOTTs at a reduced ambient temperature. We measured the trapping stiffness to examine the trap strength at different temperatures (Supplementary Note 3). Figure 2a shows the trajectories of a 1 *μ*m PS particle trapped at varying temperatures. As the temperature reduces, the particle becomes more confined with respect to the laser beam center. Figure 2b further shows the calculated trapping stiffnesses of trapped particles at varying sample temperatures with different laser powers (0.05 mW, 0.14 mW, and 0.24 mW), sizes (1 *μ*m and 9.5 *μ*m), materials (PS and SiO_2_), and solutions (DI water and electrolytes). In all the conditions, the trapping stiffness increases by 3–5 times with the reduced environmental temperature, showing the versatility of HOTTs.

To quantitatively evaluate the effect of environmental temperature on the thermophoretic behavior, we measured the drag velocity of the trapped particle to extract the Soret coefficient (*S*_*T*_) at different temperatures (Supplementary Note 4). The experimental data fit nicely with the empirical formula (Equation 2). It is noted that the Soret coefficient of the particle is tuned from 0.1 K^−1^ to −2 K^−1^ after reducing the temperature from 27 °C to 4 °C. The direction of thermophoretic force inverts and causes attraction, and the magnitude of the force increases more than 10-fold, which contributed to the increased trapping stiffness.

The thermophoretic response of the particles also depends on the colloidal concentration,^31,32^ an important parameter in drug delivery and therapeutics applications. Stable trapping at any required concentration is considered essential for bio-statistical analysis and cell-cell interactions. Here, we show that HOTTs enable the consistent trapping of colloids at concentrations spanning across several orders. As a case study, 1 *μ*m PS particles are trapped using HOTTs at varying particle concentrations and sample temperatures. The trapping performance is evaluated with the trapping probability calculated as 100×ntrap(ntrap+nrepel), where *n*_*trap*_ and *n*_*repel*_ are the number of particles that are trapped and repelled by the laser beam at the given conditions, respectively. At high colloid concentrations, the interparticle interactions stemming from surface charge, Brownian motion, and the surrounding particles’ position distribution influence the thermophoretic response of the trapped particle. Accordingly, the trapping probability fluctuates between 10–40 % at the ambient temperature (Figure 2c). As the temperature decreases, the trap probability increases and reaches 100 % at 4 °C due to the enhanced thermophilicity. At low colloid concentration, the PS particle undergoes a transition from repelling at ambient temperature to trapping at low temperatures (Figure 2d). When the temperature is reduced to 4 °C, a trapping probability of 100% is observed for all concentrations (Figure 2e), highlighting the potential applications of HOTTs for trapping in complex fluids and highly scattering media, in vitro drug efficacy, and crystallization studies.

## Trapping of erythrocytes in distinct tonicities using HOTTs

Erythrocytes (also known as erythrocyte cells or red blood cells) are important biological entities that are currently used in drug delivery^33,34^ and disease diagnostics.^35,36^ Optical trapping of erythrocytes has promoted the understanding of cell mechanics and cell-cell interactions.^37^ However, these studies are based on the local photo-deformation of the cell membrane and are limited to mature erythrocytes in isotonic solution only. Erythrocytes in different tonicities (hypertonic and hypotonic) serve as potential markers for pathophysiological disease diagnostics like sickle cell anemia^38^ and malaria.^39^ The tonicity of extracellular fluid alters the shape and size of erythrocytes, which has been recently used to determine the severity of illness caused in SARS-COV-2 patients.^40^ Although optical trapping of erythrocytes in varying tonicities can enable the extension of these cell-cell interactions and biomedical studies for diverse diseases, it is extremely challenging because of the erythrocyte’s non-linear optical response^41,42^ and photo-damage.^43^

**Figure 3:**
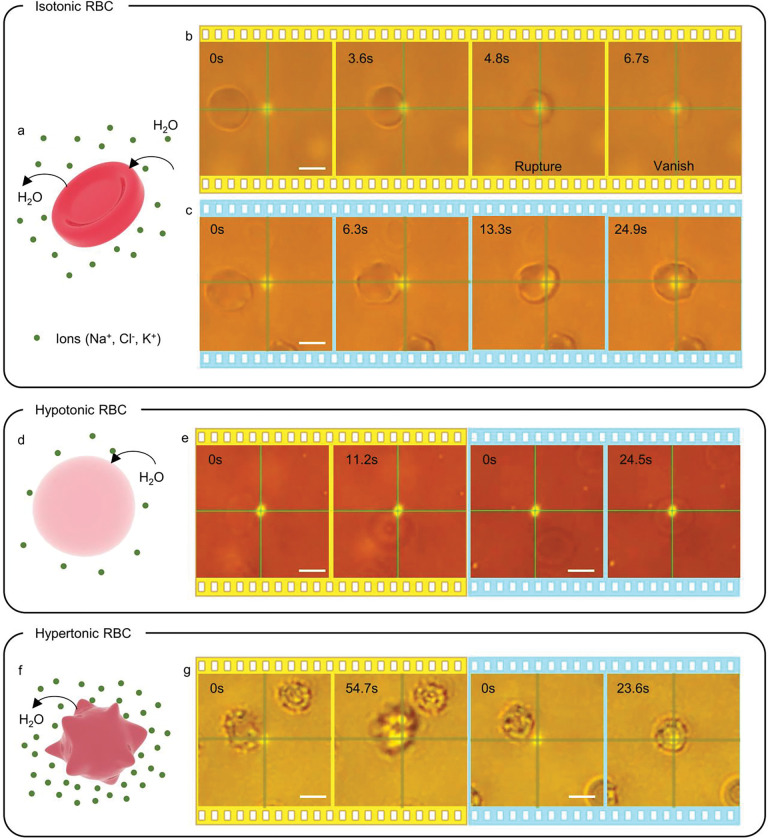
Trapping of erythrocytes in different tonicities using HOTTs: a) Schematic of erythrocytes in isotonic PBS. b) Timelapse optical images showing the trapping and thermal rupture of erythrocytes at ambient temperature. Laser power: 0.67 mW. c) Timelapse images of erythrocyte trapping at 4 °C at the same laser power. No cell rupture is observed. d) Schematic of erythrocytes in hypotonic PBS. e) Timelapse images of hypotonic erythrocytes repelled at ambient temperature (yellow panels) and trapped at 4 °C (blue panels). Laser power: 0.44 mW. f) Schematic of erythrocytes in hypertonic PBS. g) Timelapse images of hypertonic erythrocytes repelled at ambient temperature (yellow panels) and trapped at 4 °C (blue panels). Laser power: 0.34 mW. Scale bars: 5 *μ*m.

Here, we demonstrate the capability of HOTTs for biological applications by trapping erythrocytes in different tonicities at extremely low laser power, while retaining their cellular integrity and bio-physiochemical functions. The tonicity of the extracellular fluid is altered by tuning the concentration of phosphate buffered saline (PBS) solution (Figure S3). First, we dispersed healthy human blood cells in an isotonic solution, where the salinity of surrounding fluids matches that of the cells (see Methods). The cells in such an isotonic environment are disc-shaped, as shown in Figure 3a. At ambient temperature, although cells can be trapped by the laser beam, the slightly elevated temperature at the laser spot results in cell lysis. After the cell rupture, the extracellular fluid fills up the cell, and the lack of a refractive index contrast between the cell and the surrounding solution results in the instantaneous vanishing of the erythrocyte under the microscope (Figure 3b). To avoid the lysis, we reduce the sample temperature to 4 °C to stably trap the cell by HOTTs and simultaneously retain the integrity of the cellular membrane (Figure 3c, Supplementary Video 5).

When erythrocytes are dispersed in a low tonicity solution (hypotonic), the salt concentration in the extracellular fluid is lower than the erythrocyte. The osmotic gradient across the membrane results in the permeation of water into the cell and the swelling of the cell to become nearly spherical (Figure 3d). The cell is repelled by the laser at ambient temperature and trapped by the enhanced thermophoretic force at 4 °C (Figure 3e and Supplementary Video 6). Last, the high salt concentration of the extracellular fluid shrivels the erythrocyte in high tonicity solution (hypertonic) (Figure 3f). Like the hypotonic case, cells can only be trapped by HOTTs at the sub-ambient temperature (Figure 3g and Supplementary Video 7). In all tonicities, the cell integrity of erythrocytes trapped by HOTTs is maintained, indicating the good biocompatibility of HOTTs.

## 3D manipulation of plasmonic vesicles and controlled cargo release

Extracellular and synthetic vesicles have shown great importance in bioimaging, drug delivery, biological transport processes, and therapeutics.^44–46^ Plasmonic vesicles are gold-coated vesicles with controlled optical and spectroscopic properties for diverse biomedical applications.^47–50^ Although optical and optothermal trapping of naked vesicles or gold nanoparticles has been achieved,^51,52^ optical trapping of plasmonic vesicles is challenging due to the large plasmon-enhanced scattering force because of the gold layer. Also, the heat produced during laser illumination causes an uncontrollable thermophoretic force which usually directs the vesicle towards the cold (away from the laser beam) at ambient temperature.^53^ In addition, the heat generated also induces drug release from plasmonic vesicles. The capability of trapping while maintaining the integrity of plasmonic vesicles will enable precise positioning followed by optically triggered drug release, and it holds great promises in several applications.

**Figure 4:**
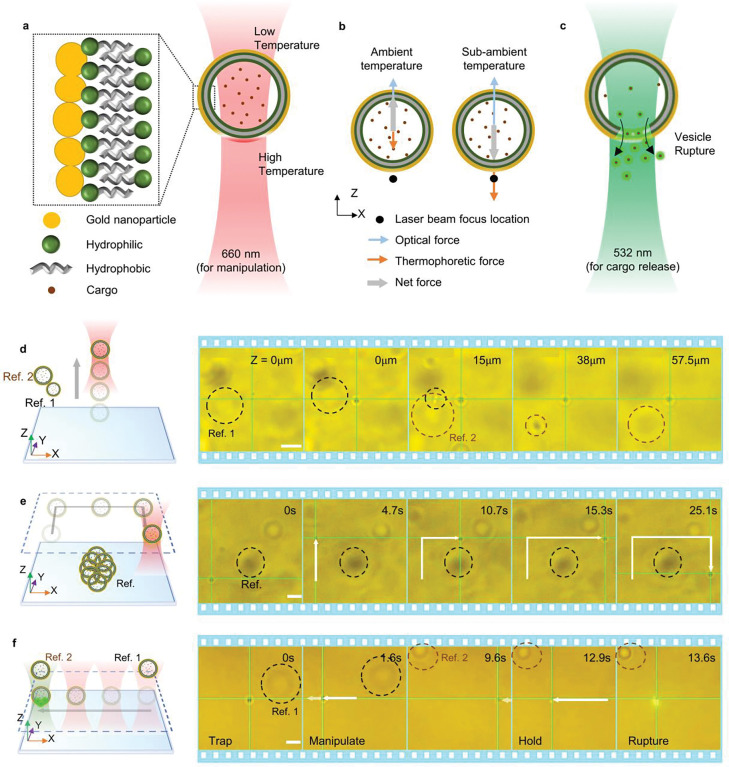
3D manipulation of plasmonic vesicles using HOTTs: a) Schematic of a plasmonic vesicle under 660 nm excitation. b) Force analysis of the plasmonic vesicle at ambient temperature and sub-ambient temperature. c) Schematic of vesicle rupture and cargo release due to 532 nm laser beam excitation (532 nm). d-f) Schematic and optical images showing (d) the levitation of a plasmonic vesicle, (e) in-plane manipulation of a plasmonic vesicle, and (f) manipulation of the vesicle and subsequent rupture for controlled cargo release. The power of the 660 nm laser for vesicle manipulation is 0.67 mW with a beam radius of 814 nm. The power of the 532 nm laser for vesicle rupture is 0.1 mW with a beam radius of 810 nm. Scale bars: 5 *μ*m.

Here, we demonstrate the trapping and 3D manipulation of plasmonic vesicles by HOTTs, followed by controlled cargo release using a dual laser beam setup. A 660 nm laser beam is utilized to manipulate the vesicle, while a 532 nm laser beam is utilized to rupture the vesicle. Under 660 nm laser irradiation, the gold coating on the plasmonic vesicles absorbs light to generate a highly localized temperature gradient across the vesicle (Figure 4a), which creates a self-induced thermophoretic force on the vesicle.^54^ Meanwhile, the high optical scattering force causes the vesicle to repel away from the focus of the laser beam at the ambient temperature irrespective of the thermophoretic force direction (Figure 4b and Supplementary Video 8). Meanwhile, the trapping of plasmonic vesicles can be achieved by HOTTs at a sub-ambient temperature of 4 °C. In this case, the self-induced thermophoretic force becomes attractive and is greatly enhanced to overcome the optical repulsion force and enable the 3D trapping of the vesicle near the focal plane (Figure 4b). After positioning the vesicle, the subsequent illumination of a 532 nm laser beam can generate intense heat to rupture the plasmonic vesicle and release the cargo (Figure 4c).

We first showed the manipulation of a plasmonic vesicle in the vertical direction by simply tuning the focus position of the laser beam (Figure 4d). The vesicle is steadily trapped and elevated for over 55 *μ*m. Next, the vesicle is transported in the lateral plane to demonstrate the in-plane manipulation (Figure 4e). We further demonstrated versatile 3D manipulation of plasmonic vesicles by HOTTs in highly complex and challenging environments (Figure S4 and Supplementary Video 9). After the vesicle is transported to the target position, a 532 nm laser beam is excited to rupture the membrane to release the cargo (Calcein dye) as shown in Figure 4f (also see Figure S5 and Supplementary Video 10). We observed an increase in fluorescent intensity since the calcein dye is self-quenched in the plasmonic vesicle and emits a stronger fluorescence upon release and dilution.

## Conclusions

We have developed HOTTs for the on-demand manipulation of diverse microparticles and biological cells by exploiting the enhanced thermophilic nature at a sub-ambient temperature. The hypothermal environmental temperature further facilitates the noninvasive trapping of fragile objects (e.g., erythrocytes) by suppressing thermal damage. This capability is desirable for a variety of biological applications, including drug development and cell-cell interactions. We further exploit HOTTs for 3D manipulation of thermosensitive plasmonic vesicles and demonstrated the light-controlled cargo release. With their versatility and general applicability, HOTTs will have many potential applications in disease diagnostics, thermal therapy, drug delivery, and microrobotic surgery.

## Methods

### Materials:

PS microparticle suspensions of 0.96 *μ*m and 2 *μ*m are purchased from Thermo Fisher Scientific. 0.5 *μ*m PS particles and 1.96 *μ*m SiO_2_ particles are purchased from Bangs Laboratories. COOH- functional PS particles of 2.67 *μ*m particles are purchased from Bangs Laboratories. Erythrocytes are purchased from Human Cells Bio and stored at 4 °C. All experiments involving erythrocytes are performed within 4 weeks of the extraction date, and new erythrocyte samples were prepared for every experiment.

### Fabrication of thermoplasmonic substrate:

Glass coverslips were triple-rinsed with iso-propyl alcohol and water and cleaned under a nitrogen gun. The coverslips were then loaded into a thermal evaporator (Kurt J Lesker Nano36), and 4.5 nm gold films are thermally deposited at a pressure of 1 × 10^−7^ torr at a rate of 0.1 nm/s. Later, gold-deposited coverslips are thermally annealed at 550 °C for two hours (ramp for 2 hours, constant temperature of 550 °C for 2 hours, and ramp for 2 hours). For microparticle experiments, thermally annealed substrates are used as prepared after cleaning using DI water and nitrogen gun. For erythrocyte experiments, the substrates are immersed in a 1mM 11-mercaptohexanoic acid in ethanol to prevent the adhesion of red blood cells onto the substrate. The modified substrates are cleaned using water droplets (gentle cleansing) to remove excess solution for uniform functional layer formation.

### Plasmonic vesicles preparation:

Plasmonic vesicles were prepared via a two-step method following the previously reported method with minor modifications.^55^ First, Dipalmitoylphosphatidylcholine (DPPC) and cholesterol in a 4:1 molar ratio were dispersed in chloroform and dried with N_2_, followed by overnight evaporation under a vacuum. The dry lipid film was then dispersed in 10 mM PBS containing calcein 75 mM for 1 hour and subsequently extruded through 400 nm polycarbonate membranes for 11 passes using Avanti Mini Extruder (Avanti Polar Lipids). Free calcein was removed by centrifugation at 5000 g for 10 mins and then washed with PBS three times. Second, gold nanoparticles were decorated onto DPPC liposome using the in-situ gold reduction method. Aqueous solutions of gold chloride (10 mM) and ascorbic acid (40 mM) were prepared. Gold chloride solution was added and gently mixed with liposome suspension (1.5 mM lipid concentration) in a molar ratio of 1:4 until uniformly distributed, followed by the addition of the same volume of ascorbic acid solution. Following reduction, plasmonic vesicles were separated from unreacted ascorbic acid and gold chloride by centrifugation (5000g, 10 mins) and then stored at 4°C until use. For trapping experiments, plasmonic vesicles that are in the sub-micrometer regime (800–1500 nm) are used due to their easy visualization under the microscope.

### Optical Setup:

For substrate-based trapping, a 532 nm laser beam (Laser Quantum Ventus 532) is passed through a 5X beam expander and directed into the objective (Nikon Plan Fluor 40x, NA 0.75) of an optical microscope (Nikon Ti-E) through a series of reflective mirrors. The liquid sample containing the target objects are loaded into a 120 *μ*m thick spacer that acts as a microchamber. A charged coupled device (CCD – Nikon DS-Fi3) is used to visualize and record particle-trapping videos. Phase camera (Phasics, SID4 Bio) is used to evaluate the temperature increase because of laser heating of the substrate. The sample is placed on an aluminum sample holder that provides edge-support on all sides of the coverslip. The Peltier thermoelectric cooler (Laird Thermal Systems SH10–23-06-L1-W4.5) along with the aluminum heat sink is then rested on top of the glass coverslips. The weight of the heat sink ensures perfect contact of the thermoelectric cooler with the sample and the temperature resistance across the interface is assumed to be negligible. An annular cooler is selected to enable the light path through the device. A corresponding through-hole is drilled into the heat sink, for white light to travel through the sample and reach the camera.

## References

[1] Li J., Hill E. H., Lin L. & Zheng Y. Optical Nanoprinting of Colloidal Particles and Functional Structures. ACS Nano 13, 3783–3795, doi:10.1021/acsnano.9b01034 (2019). 30875190 PMC6482071

[2] Li J. Opto-Thermocapillary Nanomotors on Solid Substrates. ACS Nano 16, 8820–8826, doi:10.1021/acsnano.1c09800 (2022). 35594375 PMC9949610

[3] Lei K. Cancer-cell stiffening via cholesterol depletion enhances adoptive T-cell immunotherapy. Nature Biomedical Engineering 5, 1411–1425, doi:10.1038/s41551-021-00826-6 (2021). PMC7612108 34873307

[4] Gao D. Optical manipulation from the microscale to the nanoscale: fundamentals, advances and prospects. Light: Science & Applications 6, e17039–e17039, doi:10.1038/lsa.2017.39 (2017). PMC6062326 30167291

[5] Bustamante C. J., Chemla Y. R., Liu S. & Wang M. D. Optical tweezers in single-molecule biophysics. Nature Reviews Methods Primers 1, doi:10.1038/s43586-021-00021-6 (2021). PMC8629167 34849486

[6] Corsetti S. & Dholakia K. Optical manipulation: advances for biophotonics inthe 21st century. Journal of Biomedical Optics 26, 070602, doi:10.1117/1.jbo.26.7.070602.full (2021). 34235899 PMC8262092

[7] Jiang Q., Roy P., Claude J.-B. & Wenger J. Single Photon Source from a Nanoantenna-Trapped Single Quantum Dot. Nano Letters 21, 7030–7036, doi:10.1021/acs.nanolett.1c02449 (2021). 34398613

[8] Zhang Y. Plasmonic tweezers: for nanoscale optical trapping and beyond. Light: Science & Applications 10, doi:10.1038/s41377-021-00474-0 (2021). PMC7969631 33731693

[9] Juan M. L., Gordon R., Pang Y., Eftekhari F. & Quidant R. Self-induced back-action optical trapping of dielectric nanoparticles. Nature Physics 5, 915–919, doi:10.1038/nphys1422 (2009).

[10] Pang Y. & Gordon R. Optical Trapping of 12 nm Dielectric Spheres Using Double-Nanoholes in a Gold Film. Nano Letters 11, 3763–3767, doi:10.1021/nl201807z (2011). 21838243

[11] Zhang S. Reconfigurable multi-component micromachines driven by optoelectronic tweezers. Nature Communications 12, doi:10.1038/s41467-021-25582-8 (2021). PMC8429428 34504081

[12] Chiou P. Y., Ohta A. T. & Wu M. C. Massively parallel manipulation of single cells and microparticles using optical images. Nature 436, 370–372, doi:10.1038/nature03831 (2005). 16034413

[13] Xie Y. Optoacoustic tweezers: a programmable, localized cell concentrator based on optothermally generated, acoustically activated, surface bubbles. Lab on a Chip 13, 1772, doi:10.1039/c3lc00043e (2013). 23511348 PMC3988908

[14] Lin L. Thermophoretic Tweezers for Low-Power and Versatile Manipulation of Biological Cells. ACS Nano 11, 3147–3154, doi:10.1021/acsnano.7b00207 (2017). 28230355

[15] Liu S., Lin L. & Sun H.-B. Opto-Thermophoretic Manipulation. ACS Nano 15, 5925–5943, doi:10.1021/acsnano.0c10427 (2021). 33734695

[16] Burelbach J., Zupkauskas M., Lamboll R., Lan Y. & Eiser E. Colloidal motion under the action of a thermophoretic force. The Journal of Chemical Physics 147, 094906, doi:10.1063/1.5001023 (2017). 28886631

[17] Fränzl M. & Cichos F. Hydrodynamic manipulation of nano-objects by optically induced thermo-osmotic flows. Nature Communications 13, doi:10.1038/s41467-022-28212-z (2022). PMC8813924 35115502

[18] Fränzl M. Thermophoretic trap for single amyloid fibril and protein aggregation studies. Nature Methods 16, 611–614, doi:10.1038/s41592-019-0451-6 (2019). 31235884

[19] Wang X. Graphene - Based Opto - Thermoelectric Tweezers. Advanced Materials 34, 2107691, doi:10.1002/adma.202107691 (2022). 34897844

[20] Wurger. Thermal non-equilibrium transport in colloids. Reports on Progress in Physics 73, doi:10.1088/0034-4885/73 (2010).

[21] Lin L. Opto-thermoelectric nanotweezers. Nature Photonics 12, 195–201, doi:10.1038/s41566-018-0134-3 (2018). 29785202 PMC5958900

[22] Dhont J. K. G., Wiegand S., Duhr S. & Braun D. Thermodiffusion of Charged Colloids: Single-Particle Diffusion. Langmuir 23, 1674–1683, doi:10.1021/la062184m (2007). 17279644

[23] Li J. Opto-refrigerative tweezers. Science Advances 7, eabh1101, doi:doi:10.1126/sciadv.abh1101 (2021). 34172454 PMC8232904

[24] Ahmed D. Rotational manipulation of single cells and organisms using acoustic waves. Nature Communications 7, 11085, doi:10.1038/ncomms11085 (2016). PMC4814581 27004764

[25] Gu H. Magnetic cilia carpets with programmable metachronal waves. Nature Communications 11, doi:10.1038/s41467-020-16458-4 (2020). PMC7250860 32457457

[26] Ding X. On-chip manipulation of single microparticles, cells, and organisms using surface acoustic waves. Proceedings of the National Academy of Sciences 109, 11105–11109, doi:10.1073/pnas.1209288109 (2012). PMC3396524 22733731

[27] Trivedi M., Saxena D., Ng W. K., Sapienza R. & Volpe G. Self-organized lasers from reconfigurable colloidal assemblies. Nature Physics 18, 939–944, doi:10.1038/s41567-022-01656-2 (2022).

[28] Duhr S. & Braun D. Why molecules move along a temperature gradient. Proceedings of the National Academy of Sciences 103, 19678–19682, doi:10.1073/pnas.0603873103 (2006). PMC1750914 17164337

[29] Sehnem A. L. Temperature dependence of the Soret coefficient of ionic colloids. Physical Review E 92, doi:10.1103/physreve.92.042311 (2015). 26565244

[30] Thermophoresis in protein solutions. Europhysics Letters 63, 247–253, doi:10.1209/epl/i2003 (2003).

[31] Jiang H.-R., Wada H., Yoshinaga N. & Sano M. Manipulation of Colloids by a Nonequilibrium Depletion Force in a Temperature Gradient. Physical Review Letters 102, doi:10.1103/physrevlett.102.208301 (2009). 19519079

[32] Niether D. & Wiegand S. Heuristic Approach to Understanding the Accumulation Process in Hydrothermal Pores. Entropy 19, 33, doi:10.3390/e19010033 (2017).

[33] Brenner J. S. Red blood cell-hitchhiking boosts delivery of nanocarriers to chosen organs by orders of magnitude. Nature Communications 9, doi:10.1038/s41467-018-05079-7 (2018). PMC6041332 29992966

[34] Burns J. M., Vankayala R., Mac J. T. & Anvari B. Erythrocyte-Derived Theranostic Nanoplatforms for Near Infrared Fluorescence Imaging and Photodestruction of Tumors. ACS Applied Materials & Interfaces 10, 27621–27630, doi:10.1021/acsami.8b08005 (2018). 30036031 PMC6526021

[35] Turlier H. Equilibrium physics breakdown reveals the active nature of red blood cell flickering. Nature Physics 12, 513–520 (2016).

[36] Agrawal R. Assessment of red blood cell deformability in type 2 diabetes mellitus and diabetic retinopathy by dual optical tweezers stretching technique. Scientific Reports (2017). 10.1038/srep15873 PMC4792142 26976672

[37] Zhu R., Avsievich T., Popov A. & Meglinski I. Optical Tweezers in Studies of Red Blood Cells. Cells 9, 545, doi:10.3390/cells9030545 (2020). 32111018 PMC7140472

[38] Carden M. A. Extracellular fluid tonicity impacts sickle red blood cell deformability and adhesion. Blood 130, 2654–2663, doi:10.1182/blood-2017-04-780635 (2017). 28978568 PMC5731085

[39] Duez J. High-throughput microsphiltration to assess red blood cell deformability and screen for malaria transmission–blocking drugs. Nature Protocols 13, 1362–1376, doi:10.1038/nprot.2018.035 (2018). 29844524

[40] Lee J. J. Association between red blood cell distribution width and mortality and severity among patients with COVID-19: A systematic review and meta-analysis. Journal of Medical Virology 93, 2513–2522, doi:10.1002/jmv.26797 (2021). 33448439 PMC8014709

[41] Li Y. Trapping and Detection of Nanoparticles and Cells Using a Parallel Photonic Nanojet Array. ACS Nano 10, 5800–5808, doi:10.1021/acsnano.5b08081 (2016). 27163754

[42] Gautam R. Optical force-induced nonlinearity and self-guiding of light in human red blood cell suspensions. Light: Science & Applications 8, doi:10.1038/s41377-019-0142-1 (2019). PMC6414597 30886708

[43] Blázquez-Castro A. Optical Tweezers: Phototoxicity and Thermal Stress in Cells and Biomolecules. Micromachines 10, 507, doi:10.3390/mi10080507 (2019). 31370251 PMC6722566

[44] El Andaloussi S., Mäger I., Breakefield X. O. & Wood M. J. A. Extracellular vesicles: biology and emerging therapeutic opportunities. Nature Reviews Drug Discovery 12, 347–357, doi:10.1038/nrd3978 (2013). 23584393

[45] Pan S. Extracellular vesicle drug occupancy enables real-time monitoring of targeted cancer therapy. Nature Nanotechnology 16, 734–742, doi:10.1038/s41565-021-00872-w (2021). 33686255

[46] Herrmann I. K., Wood M. J. A. & Fuhrmann G. Extracellular vesicles as a next-generation drug delivery platform. Nature Nanotechnology 16, 748–759, doi:10.1038/s41565-021-00931-2 (2021). 34211166

[47] Xiong H. Near-Infrared Light Triggered-Release in Deep Brain Regions Using Ultra - photosensitive Nanovesicles. Angewandte Chemie International Edition 59, 8608–8615, doi:10.1002/anie.201915296 (2020). 32124529 PMC7362956

[48] Gorgoll R. M., Tsubota T., Harano K. & Nakamura E. Cooperative Self-Assembly of Gold Nanoparticles on the Hydrophobic Surface of Vesicles in Water. Journal of the American Chemical Society 137, 7568–7571, doi:10.1021/jacs.5b03632 (2015). 26043281

[49] Song J., Huang P., Duan H. & Chen X. Plasmonic Vesicles of Amphiphilic Nanocrystals: Optically Active Multifunctional Platform for Cancer Diagnosis and Therapy. Accounts of Chemical Research 48, 2506–2515, doi:10.1021/acs.accounts.5b00059 (2015). 26134093 PMC5242367

[50] Xiong H. Probing Neuropeptide Volume Transmission In Vivo by Simultaneous Near - Infrared Light - Triggered Release and Optical Sensing**. Angewandte Chemie International Edition 61, doi:10.1002/anie.202206122 (2022). PMC9388559 35723610

[51] Bolognesi G. Sculpting and fusing biomimetic vesicle networks using optical tweezers. Nature Communications 9, doi:10.1038/s41467-018-04282-w (2018). PMC5951844 29760422

[52] Hill E. H., Li J., Lin L., Liu Y. & Zheng Y. Opto-Thermophoretic Attraction, Trapping, and Dynamic Manipulation of Lipid Vesicles. Langmuir 34, 13252–13262, doi:10.1021/acs.langmuir.8b01979 (2018). 30350700 PMC6246038

[53] Talbot E. L., Kotar J., Parolini L., Di Michele L. & Cicuta P. Thermophoretic migration of vesicles depends on mean temperature and head group chemistry. Nature Communications 8, 15351, doi:10.1038/ncomms15351 (2017). PMC5512737 28513597

[54] Lin L. Opto-thermoelectric pulling of light-absorbing particles. Light: Science & Applications 9, doi:10.1038/s41377-020-0271-6 (2020). PMC7058623 32194948

[55] Li X., Che Z., Mazhar K., Price T. J. & Qin Z. Ultrafast Near-Infrared Light-Triggered Intracellular Uncaging to Probe Cell Signaling. Advanced Functional Materials 27, 1605778, doi:10.1002/adfm.201605778 (2017). 29176940 PMC5697715

